# Identification of key genes increasing susceptibility to atrial fibrillation in nonalcoholic fatty liver disease and the potential mechanisms: mitochondrial dysfunction and systemic inflammation

**DOI:** 10.3389/fphar.2024.1360974

**Published:** 2024-03-14

**Authors:** Baiyin Zhong, Zhonghui Xie, Jianhong Zhang, Xing Xie, Yuankang Xie, Binhui Xie, Jing Wang, Chuanbin Liu

**Affiliations:** ^1^ Department of Hepatobiliary Surgery, The First Affiliated Hospital of Gannan Medical University, Ganzhou, China; ^2^ Department of Cardiology, Tianjin Medical University General Hospital, Tianjin, China; ^3^ Department of General Medicine, The First Medical Center of PLA General Hospital, Beijing, China; ^4^ Western Medical Branch of PLA General Hospital, Beijing, China

**Keywords:** non-alcoholic fatty liver disease, atrial fibrillation, mitochondrial dysfunction, systemic inflammation, empagliflozin

## Abstract

**Background:** Non-alcoholic fatty liver disease (NAFLD) and atrial fibrillation (AF) are major health burdens, with emerging evidence suggesting NAFLD as a significant risk factor for AF, but the mechanism is remain unclear.

**Methods:** In this study, we analyzed gene expression data from NAFLD (GSE89632) and AF (GSE75092) datasets from the Gene Expression Omnibus. We identified co-upregulated and co-downregulated genes between NAFLD and AF, assessed diagnostic potential of specific genes, conducted immune infiltration analysis, and performed molecular docking studies with sodium glucose co-transporter 2 inhibitors (SGLT2i).

**Results:** We identified eight co-upregulated and 31 co-downregulated genes between NAFLD and AF. Genes such as *AMOT*, *PDE11A*, *TYMS*, *TMEM98*, and *PTGS2* demonstrated substantial diagnostic potential for identifying NAFLD patients at risk of AF. Immune infiltration analysis discovered an elevated presence of CD8 T cells, γδ T cells, and M2 macrophages in NAFLD livers, linking systemic inflammation to NAFLD and AF. Additionally, studies have shown that a connection between mitochondrial dysfunction and several hub genes like *DGAT1*, *TYMS*, and *PTGS2*, suggesting that mitochondrial disturbances may underpin the systemic inflammation in NAFLD, which possibly exacerbating AF. Molecular docking studies indicated that empagliflozin's binding affinity with key genes such as *DGAT1*, *TYMS*, and *PTGS2* presents a novel therapeutic avenue for NAFLD-associated AF.

**Conclusion:** Our study firstly discovered that *AMOT, PDE11A, TYMS, TMEM98*, and *PTGS2* are associated with NAFLD-related AF and hold strong diagnostic values. Our study also indicates that mitochondrial dysfunction and systemic inflammation may be potential mechanisms bridging NAFLD and AF. Additionally, we identified empagliflozin as a potentially effective therapeutic agent for NAFLD-related AF at the molecular structure level. These novel insights contribute to the further understanding, diagnosis, and intervention of NAFLD-related AF.

## 1 Introduction

Atrial fibrillation (AF) and non-alcoholic fatty liver disease (NAFLD) exhibit substantial epidemiological prevalence and impact on health. AF, known for its high mortality, disability, and recurrence rates, heightens the risk of stroke, myocardial infarction, and heart failure (Joseph et al., 2021). Concurrently, NAFLD is globally the leading liver and metabolic disorder (Zhou et al., 2020). By 2018, nearly 25% of the world population, i.e., 1.7 billion people, were affected by NAFLD (Cai et al., 2019b), exceeding the combined total of obese (0.65 billion) and diabetic (0.4 billion) populations (The Lancet Public, 2018). Research has demonstrated a solid link between NAFLD and elevated AF risk, independent of other traditional cardio-metabolic comorbidities (Chen et al., 2021). NAFLD’s contribution to AF onset and recurrence post-ablation is evident, even when adjusted for age, sex, body mass index (BMI), ejection fraction, and AF type (Donnellan et al., 2020). However, despite its recognition as a new cardiac arrhythmia risk factor, NAFLD-related AF remains under-explored, and its underlying mechanisms are unclear.

Emerging studies have shed light on pathophysiological alterations associated with NAFLD that could induce cardiac structural, electrical, and autonomic neural remodeling, potentially leading to cardiac arrhythmias, with some highlighting the key role of the liver-heart inflammation axis in hepatogenic cardiomyopathy development (Matyas et al., 2020). Nevertheless, NAFLD’s role as a novel arrhythmia risk factor is largely underestimated, underplaying its impact on patient morbidity. For example, systemic inflammation, a hallmark of NAFLD, could contribute to atrial structural remodeling, a known AF precursor (Cai et al., 2019a). Metabolic stress in NAFLD leads to hepatic lipid accumulation and subsequent lipotoxicity, with the release of danger-associated molecular patterns, while recent insights point to mitochondrial dysfunction—marked by disrupted energy metabolism and increased oxidative stress—as a factor that not only exacerbates systemic inflammation but also may underlie the arrhythmic tendencies observed in AF (Liu et al., 2020). These molecules may trigger sterile inflammation pathways, boosting local inflammatory responses. The activation of hepatic immune signaling results in the release of pro-inflammatory cytokines and chemokines (Zhang et al., 2017). Importantly, these inflammatory mediators can enter the systemic circulation via the hepatic sinusoidal blood flow, augmenting a systemic pro-inflammatory environment (Cai et al., 2018). Elevated systemic inflammatory markers and inducers have been identified in both NAFLD patients and experimental models (Mohammed et al., 2021; Wang et al., 2021). Research also shows associations between hepatic fat and inflammatory markers, independent of obesity and diabetes, suggesting that NAFLD could directly trigger immune activation and systemic inflammation, possibly affecting distant organs like the heart, thereby increasing AF susceptibility (Käräjämäki et al., 2018). Additionally, the convergence of systemic inflammation and mitochondrial dysfunction in NAFLD may amplify the risk of developing AF, indicating a potential target for therapeutic intervention. Sodium glucose co-transporter 2 inhibitor (SGLT2i) is a new type of hypoglycemic agent, which also shows good application prospects in the cardiovascular diseases (Panico et al., 2023). In recent years, numerous studies have confirmed that SGLT2i could reduce the risk of new-onset AF in patients with Type 2 diabetes mellitus (T2DM), increase the clinical benefit of patients with T2DM combined with AF, and reduce the recurrence of AF after AF ablation (Zinman et al., 2015; Zelniker et al., 2020; Zhao et al., 2023). NAFLD and T2DM are metabolic abnormalities with many common pathophysiological bases, such as glucose metabolism abnormality, insulin resistance, systemic inflammation, and mitochondrial dysfunction (Muzurović et al., 2021; Kosmalski et al., 2023). Studies have shown that SGLT2i could improve not only glycemic control but also liver fatty infiltration and fibrosis in patients with NAFLD and T2DM (Arai et al., 2021; Akuta et al., 2022). In addition, recent studies also found that the direct effect of SGLT2i on NAFLD, that SGLT2i could attenuate high-fat diet-induced steatosis in mice (Nasiri-Ansari et al., 2021; Radlinger et al., 2023). Therefore, we hypothesized that SGLT2i may be a potential therapeutic agent for NAFLD-related AF.

CIBERSORT, a widely used tool for deconvoluting human immune cell subtype expression matrices based on linear support vector regression, provides gene expression feature sets for 22 immune cell subtypes using known reference datasets: LM22 (Newman et al., 2015). CIBERSORT has been primarily used in cancer, but recent years have seen its application in non-cancerous diseases like atherosclerosis, myocardial infarction, and ischemic stroke (Huang B. et al., 2022; Xie et al., 2023). However, CIBERSORT’s use in NAFLD and AF is less frequent, with no studies reporting the immune profile of NAFLD-related AF patients.

In this study, we aim to analyze the common genes in NAFLD and AF patients from the GEO database and validate our findings with reverse transcription-quantitative polymerase chain reaction (RT-qPCR) on patients’ peripheral blood leukocyte samples. We then performed immune infiltration analysis with CIBERSORT on tissue microarrays for each condition, selecting genes associated with specific immune cells. Last but not least, we explored the theoretical feasibility of SGLT2i for the treatment of NAFLD-related AF by molecular docking studies. Our ultimate goal is to find the association between NAFLD and AF, especially the possible mechanisms by which NAFLD increases susceptibility to AF, and identify biological markers and potential targets for intervention associated with NAFLD-related AF.

## 2 Materials and methods

**FIGURE 1:**
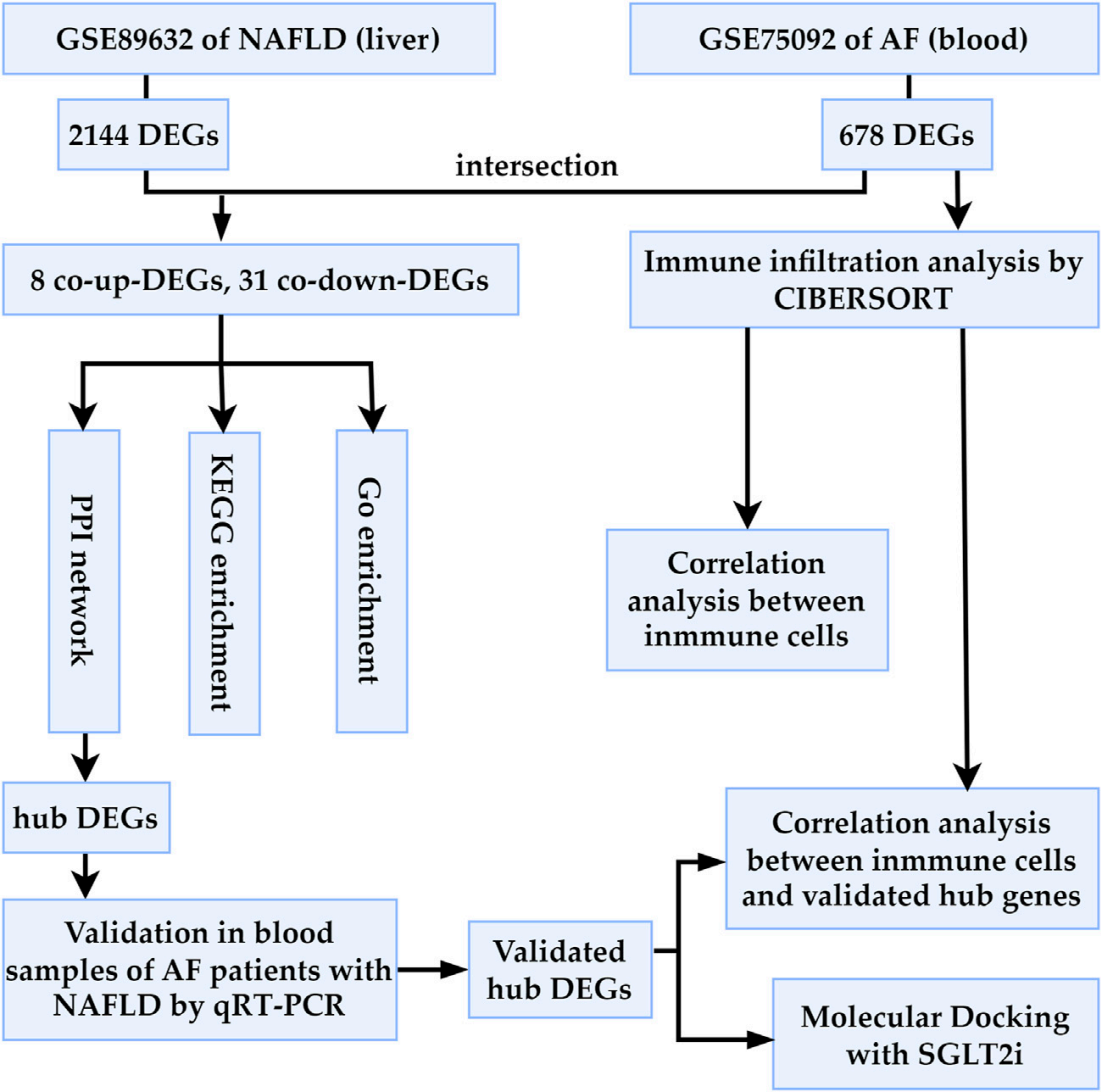
Workflow of the bioinformatics analysis methods in the present study. AF, atrial fibrillation; NAFLD, nonalcoholic fatty liver disease; DEGs, differentially expressed genes; SGLT2i, sodium glucose co-transporter 2 inhibitor.

We retrieved datasets GSE89632 and GSE75092 from the Gene Expression Omnibus (GEO) database (http://www.ncbi.nlm.nih.gov/geo/). GSE89632 (platform: GPL14951) includes liver tissue specimens from 24 healthy controls (HC) and 39 NAFLD patients. GSE75092 (platform: GLP16956) comprises blood samples from 3 AF patients and 3 sinus rhythm (SR) controls. As shown in Figure 1, we first calculated the differentially expressed genes (DEGs) of GSE89632 and GSE75092, then study the potential relationship between NAFLD and AF. The HC presence of a normal liver (no steatosis or cirrhosis) on imaging and/or histology, while the NAFLD patients were diagnosed by liver biopsy, as indicated elsewhere (Arendt et al., 2015). The AF patients in this study were all diagnosed with paroxysmal AF by electrocardiograph (ECG), whereas the SR patients had no clinical evidence or history of AF (Su et al., 2018).

### 2.1 Data processing and identification of DEGs and Co-DEGs

The raw datasets of GSE89632 and GSE75092 were processed using R (Version 4.3.0) packages, including “affy,” “affyPLM,” and “limma,” for background correction and normalization. To filter out DEGs, we set the thresholds as an adjusted *p*-value <0.05 and |log2FC| < 0.585. Volcano plots, generated using the “ggplot2” and “ggrepel” packages, visualized mRNA expression levels and corresponding statistical inference values. Venn diagrams were constructed using the “VennDiagram” package to identify co-DEGs for both NAFLD and AF.

### 2.2 Functional enrichment analysis

We performed GO and KEGG pathway enrichment analyses for co-DEGs using the “clusterProfiler” and “pathview” packages in R software. Enrichment results were displayed using bar graphs, with a q-value (adjusted *p*-value) threshold of 0.05.

### 2.3 Protein-protein interaction network

The PPI network of co-DEGs was analyzed using the STRING database (Version 11.5; http://string-db.org/), and the results were visualized in Cytoscape software (Version 3.7.2). The biological networks and node degrees were graphically represented, and the top ten hub genes were identified.

### 2.4 Validation for the potential role of hub genes

The study protocols and the use of human blood samples were following the Declaration of Helsinki and were approved by the Human Ethics Review Committee of the Chinese PLA General Hospital. Written informed consent was obtained from all patients/participants. AF patients were diagnosed using ECG, while SR patients did not exhibit any AF symptoms or ECG evidence. The HC group included participants confirmed to have no liver diseases through abdominal ultrasonography, indicating no hepatic lesions. NAFLD patients were diagnosed using abdominal ultrasound. Leukocyte samples were obtained from the PLA General Hospital Biosample Bank, including 15 HC, 15 AF, 15 NAFLD, and 15 NAFLD-AF patients (Table 1). Leukocytes were isolated from human peripheral blood using Ficoll density gradient centrifugation according to the manufacturer’s instructions and stored at −80°C until further analysis. Detailed patient characteristics are summarized in Table 1. Total RNA from blood was extracted using a high-performance blood total RNA extraction kit (DP443, TIANGEN) following the standard protocol. Reverse transcription of RNA was performed using a reverse transcription system (K1622, Thermo Fisher, Waltham, MA, United States). The obtained cDNA was amplified using the SYBR premix kit (A25742, Thermo Fisher) with BIO-RAD CFX96 (Bio-Rad Laboratories, Hercules, CA, United States). RT-qPCR was conducted as follows: pre-denaturation at 95°C for the 30 s; denaturation at 95°C for 5 s, followed by 40 cycles at 60°C for 30–34 s. All experiments were performed in triplicates. Pre-designed gene-specific primers are listed in Supplementary Table S1, and the housekeeping gene GAPDH was used as the endogenous reference. The relative mRNA expression levels were calculated using the 2^−ΔΔCt^ method.

**TABLE 1:** Patients’ baseline characteristics.

	HC	AF	NAFLD	NAFLD-AF
Number	15	15	15	15
Male n (%)	7 (46.67%)	7 (46.67%)	7 (46.67%)	7 (46.67%)
Age (in years)	59.80% ± 2.68%	60.60% ± 5.18%	66.20% ± 7.33%	63.60% ± 5.32%
BMI (kg/m^2^)	23.62% ± 1.98%	22.88% ± 2.66%	24.18% ± 0.96%	25.15% ± 1.01%
Diabetes n (%)	4 (26.67%)	6 (40.00%)	3 (20.00%)	6 (40.00%)
Hyperlipidemia n (%)	3 (20.00%)	5 (33.33%)	3 (20.00%)	6 (40.00%)
Hypertension n (%)	5 (33.33%)	6 (40.00%)	6 (40.00%)	8 (53.33%)

Values are mean ± SEM or numbers (percentage). BMI, body mass index; HC, healthy control; AF, atrial fibrillation; NAFLD, nonalcoholic fatty liver disease.

### 2.5 ROC curve plotting

Data analysis was performed using the R statistical software (Version 4.3.0). Specifically, the “pROC” package (Version 1.17.0.1) was used to generate receiver operating characteristic (ROC) curves for the gene expression data. Plots of the ROC curves were generated using the “ggplot2” package (Version 3.3.5) in R. Each plot displayed the sensitivity (true positive rate) on the Y-axis and 1-specificity (false positive rate) on the X-axis. AUC values were also included in the plot for a comprehensive representation of the discriminatory power of each gene. Statistical significance of the AUC values was assessed using DeLong’s test, a non-parametric method for comparing two ROC curves, which is incorporated into the “roc.test” function in the “pROC” package.

### 2.6 Immune infiltration analysis by CIBERSORT

We employed the CIBERSORT algorithm to examine the normalized data derived from raw data, utilizing the LM22 as a reference gene matrix (Newman et al., 2015). LM22 includes macrophages M0, M1, and M2, memory B cells, B naïve cells, plasma cells, CD8 T cells, gamma-delta T cells, follicular helper T cells, CD4 memory-activated T cells, CD4 naïve T cells, CD4 memory resting T cells, regulatory T cells (Tregs), neutrophils, monocytes, activated NK cells, resting NK cells, resting dendritic cells, activated dendritic cells, eosinophils, activated mast cells, and resting mast cells. Samples with a *p*-value below 0.05 were deemed successful for deconvolution analysis and further differential analysis of immune cell proportions. The “ggplot2,” “pheatmap,” and “vioplot” packages facilitated the visualization of immune cell infiltration percentage and differences. Relationships between immune cells and validated hub genes were computed using the “corrplot” package.

### 2.7 Molecular docking studies

In this study, we employed molecular docking techniques to investigate the interactions between three currently the most commonly used SGLT2i (empagliflozin, dapagliflozin, and canagliflozin) and three distinct protein targets (DGAT1, TYMS, and PTGS2). The structural data for these target proteins were retrieved from the Protein Data Bank (PDB, https://www.rcsb.org/), with the respective PDB IDs being 6VYI, 6ZXO, and 5F19. The chemical structures of small drug molecules were retrieved from the PubChem database (https://pubchem.ncbi.nlm.nih.gov/). Initially, the three-dimensional (3D) structures of the SGLT2i were analyzed, and any water molecules and organic solvents associated with the protein structures were removed using PyMOL software. Subsequently, the active sites of the proteins were identified using AutoDock Tools (ADT), setting the stage for the docking simulations. The 3D conformations of the SGLT2 inhibitors, converted from their two-dimensional (2D) structures within ChemOffice, were optimized through energy minimization employing the MM2 force field. This step ensured the geometric configurations of the ligands were ideal for interaction. Molecular docking simulations were then conducted with AutoDock Vina, focusing on the predetermined active sites facilitated by ADT. Visualization and analysis of the optimal docking poses were achieved using PyMOL software, which allowed for an in-depth examination of the interaction modes between the SGLT2 inhibitors and their respective protein targets.

### 2.8 Statistical analysis

R (4.3.0) facilitated bioinformatics analysis and ROC plotting. PASS (version 15.0; NCSS, United States) was utilized for sample size analysis. For qRT-PCR data, we used SPSS statistical package (version 21.0; SPSS Inc., Chicago, United States) for statistical analyses, employing one-way ANOVA and chi-square tests to determine group differences. Both an adjusted *p*-value (q-value) and a *p*-value under 0.05 were deemed statistically significant.

## 3 Results

### 3.1 Identification of DEGs in NAFLD and AF

**FIGURE 2:**
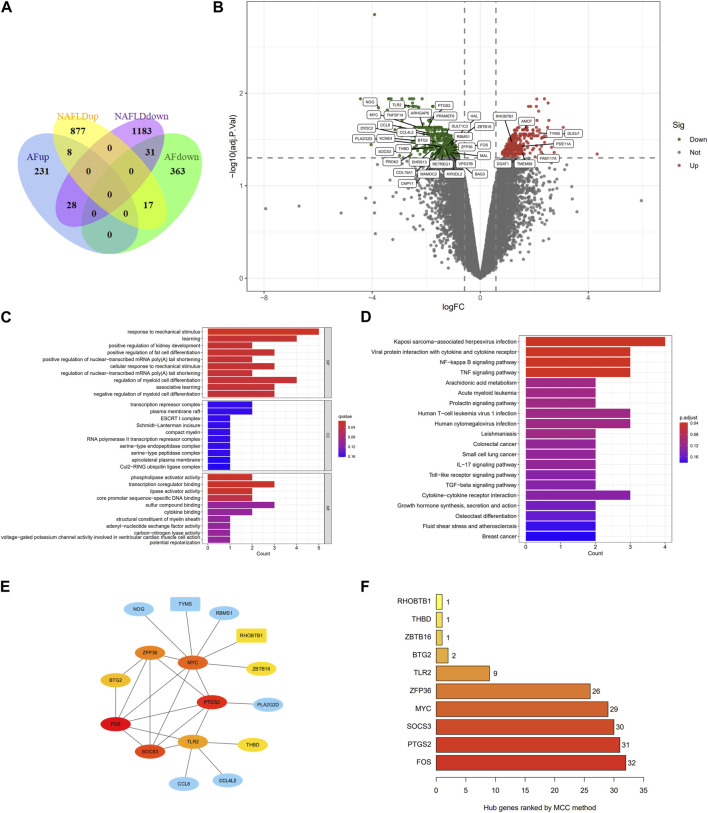
Venn diagram, volcano plot, GO term enrichment, KEGG pathway enrichment, PPI network, and hub genes of AF- and NAFLD-related DEGs. **(A)** Venn diagram showcasing DEGs associated with AF and NAFLD. **(B)** Volcano plot of AF-related DEGs. Gray points correspond to adjusted *p* > 0.05; red points denote upregulated genes with adjusted *p* < 0.05; green points represent downregulated genes with adjusted *p* < 0.05. **(C)** GO term enrichment for co-down-regulated DEGs linked to AF and NAFLD. Dot size indicates the count of enriched DEGs; dot color reflects the q value. **(D)** KEGG pathway of co-DEGs related to AF and NAFLD. Dot size denotes the count of enriched DEGs; dot color illustrates the q value. **(E)** Cytoscape software calculated the hub genes of co-DEGs. Red indicates a higher MCC score; blue signifies a lower MCC score. Rectangles represent co-up-regulated DEGs; ellipses correspond to co-down-regulated DEGs. **(F)** Hub genes of co-DEGs are ranked by the MCC method. GO, Gene Ontology; KEGG, Kyoto Encyclopedia of Genes and Genomes; PPI, protein-protein interaction; AF, atrial fibrillation; NAFLD, nonalcoholic fatty liver disease; DEGs, differentially expressed genes.

In the GSE89632 dataset, we identified 29,378 probes corresponding to 18,991 genes, and in the GSE75092 dataset, we detected 73,927 probes for 15,918 genes. A total of 2,144 DEGs were identified between NAFLD patients and healthy controls (HC) in GSE89632 liver specimens, comprising 902 upregulated and 1,242 downregulated genes. Meanwhile, 678 DEGs were identified between AF patients and SR patients in GSE75092 blood specimens, including 411 upregulated and 267 downregulated genes. All data is available in Supplementary Table S2. Heatmaps for the top 50 upregulated and downregulated genes for both NAFLD-DEGs and AF-DEGs can be found in Supplementary Figures S1, S2. Figure 2A provides a visual depiction of the number of co-expressed genes between NAFLD- and AF-DEGs, with the gene distribution displayed in Figure 2B.

### 3.2 GO terms, KEGG pathway enrichment in Co-DEGs

Given that eight co-upregulated DEGs were not enriched in gene ontology (GO) terms and Kyoto Encyclopedia of Genes and Genomes (KEGG) pathway, only the enrichment of 31 downregulated genes was shown in Figure 2C; Supplementary Table S3. The top ten GO terms related to biological processes (BPs) among these genes were: response to mechanical stimulus (GO:0009612), learning (GO:0007612), positive regulation of kidney development (GO:0090184), positive regulation of fat cell differentiation (GO:0045600), positive regulation of nuclear-transcribed mRNA poly (A) tail shortening (GO:0060211), cellular response to mechanical stimulus (GO:0071260), regulation of nuclear-transcribed mRNA poly(A) tail shortening (GO:0060211), regulation of myeloid cell differentiation (GO:0045637), and associative learning (GO:0008306). In terms of cellular components (CCs), there was no significant enrichment. The terms related to molecular function (MF) mainly include phospholipase activator activity (GO:0016004), transcription coregulator binding (GO:0001221), lipase activator activity (GO:0060229), core promoter sequence-specific DNA binding (GO:0001046).

The KEGG pathway enrichment results are shown in Figure 2D; Supplementary Table S3. In the KEGG analysis, the downregulated genes were mainly enriched in Kaposi sarcoma-associated herpesvirus infection (hsa05167), Viral protein interaction with cytokine and cytokine receptor (hsa04061), NF-kappa B signaling pathway (hsa04064), and TNF signaling pathway (hsa04668).

### 3.3 PPI network analysis in Co-DEGs

39 co-DEGs were incorporated into the protein-protein interaction (PPI) network, yielding 16 nodes and 25 edges (Supplementary Figure S3). The hub genes calculated by maximal clique centrality (MCC) algorithm with Cytoscape software are FOS (score 32), PTGS2 (score 31), SOCS3 (score 30), MYC (score 29), ZFP36 (score 26), TLR2 (score 9), BTG2 (score 2), ZBTB16 (score 1), THBD (score 1), and RHOBTB1 (score 1), which are considered to be associated with NAFLD-related AF (Figures 2E, F).

### 3.4 Validation for the potential role of co-DEGs

**FIGURE 3:**
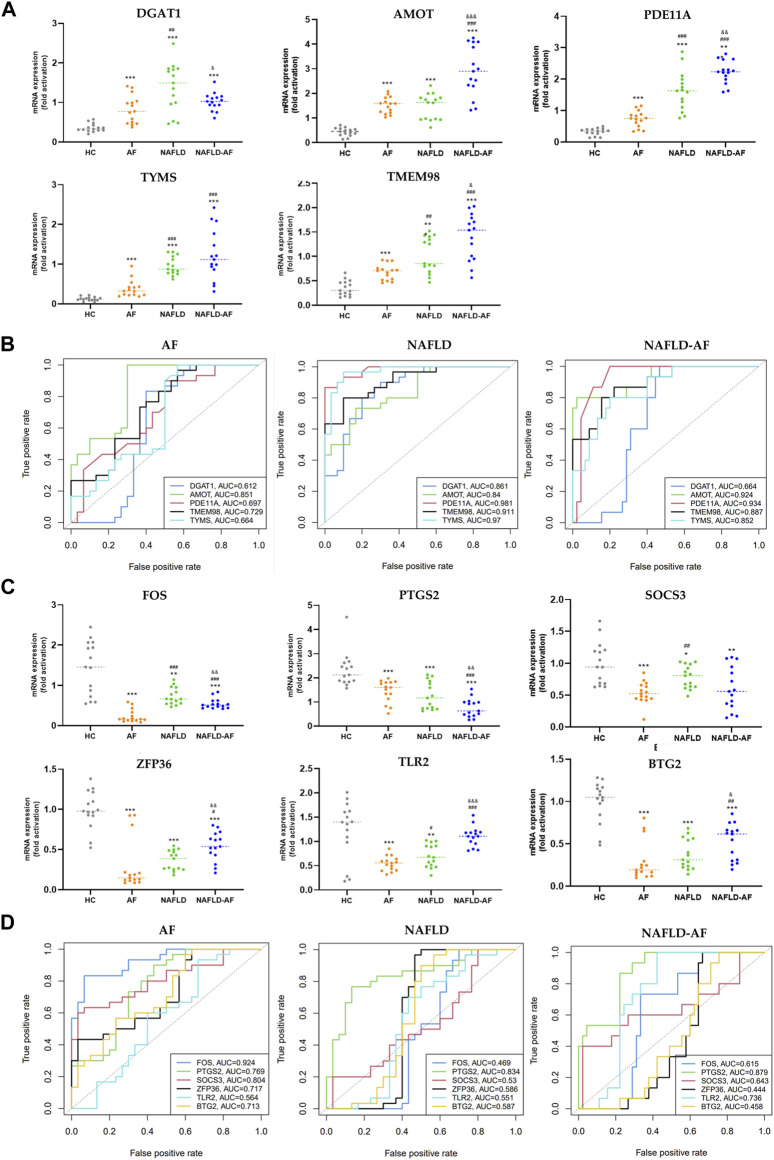
Validation of hub genes. **(A)** Validation of co-upregulated DEGs in patients’ blood cells by RT-qPCR. **(B)** ROC curve for validated co-upregulated-DEGs. **(C)** Validation of co-downregulated-DEGs in patients’ blood cells by RT-qPCR. **(D)** ROC curve for validated co-downregulated-DEGs. (* compared with HC; # compared with AF; & compared with NAFLD; *^/#/&^ *p* < 0.05, **^/##/&&^ *p* < 0.01, ***^/###/&&&^ *p* < 0.001). AF, atrial fibrillation; NAFLD, nonalcoholic fatty liver disease; HC, healthy control; DEGs, differentially expressed genes; ROC, receiver operator characteristic.

Based on a comprehensive review of the literature, previous results from enrichment analysis, and PPI data, we validated five co-upregulated genes (DGAT1, AMOT, PDE11A, TYMS, and TMEM98) and six co-downregulated genes (FOS, PTGS2, SOCS3, ZFP36, TLR2, and BTG2) in the blood samples of patients from four groups: HC, AF, NAFLD, and NAFLD-AF. As depicted in Figure 3A, DGAT1, AMOT, PDE11A, TYMS, and TMEM98 were found to be higher in the AF group, NAFLD group, and NAFLD-AF group compared to the HC group. However, AMOT, PDE11A, TYMS, and TMEM98 were distinctly expressed at elevated levels in the NAFLD-AF group compared to the other three groups of patients. Similarly, as illustrated in Figure 3C, the expression of FOS, PTGS2, SOCS3, ZFP36, and BTG2 was higher in the AF group, NAFLD group, and NAFLD-AF group compared to the HC group, whereas PTGS2 expression was notably lower in the NAFLD-AF group.

Moreover, as shown in Figures 3B, D, AMOT, PDE11A, TYMS, TMEM98, and PTGS2 demonstrated significant diagnostic efficacy for NAFLD-associated AF. The area under the curve (AUC), is denoted as AMOT (0.924, 0.841–1), PDE11A (0.934, 0.870–0.998), TYMS (0.952, 0.748–0.956), TMEM98 (0.887, 0.793–0.982), and PTGS2 (0.879, 0.790–0.967), respectively. These results suggesting these genes could serve as potential biomarkers for differentiating the NAFLD-AF group from HC, AF, and NAFLD patients. Further research is needed to fully understand the roles of these genes in the pathogenesis and progression of NAFLD-related AF.

### 3.5 Immune infiltration analyses

**FIGURE 4:**
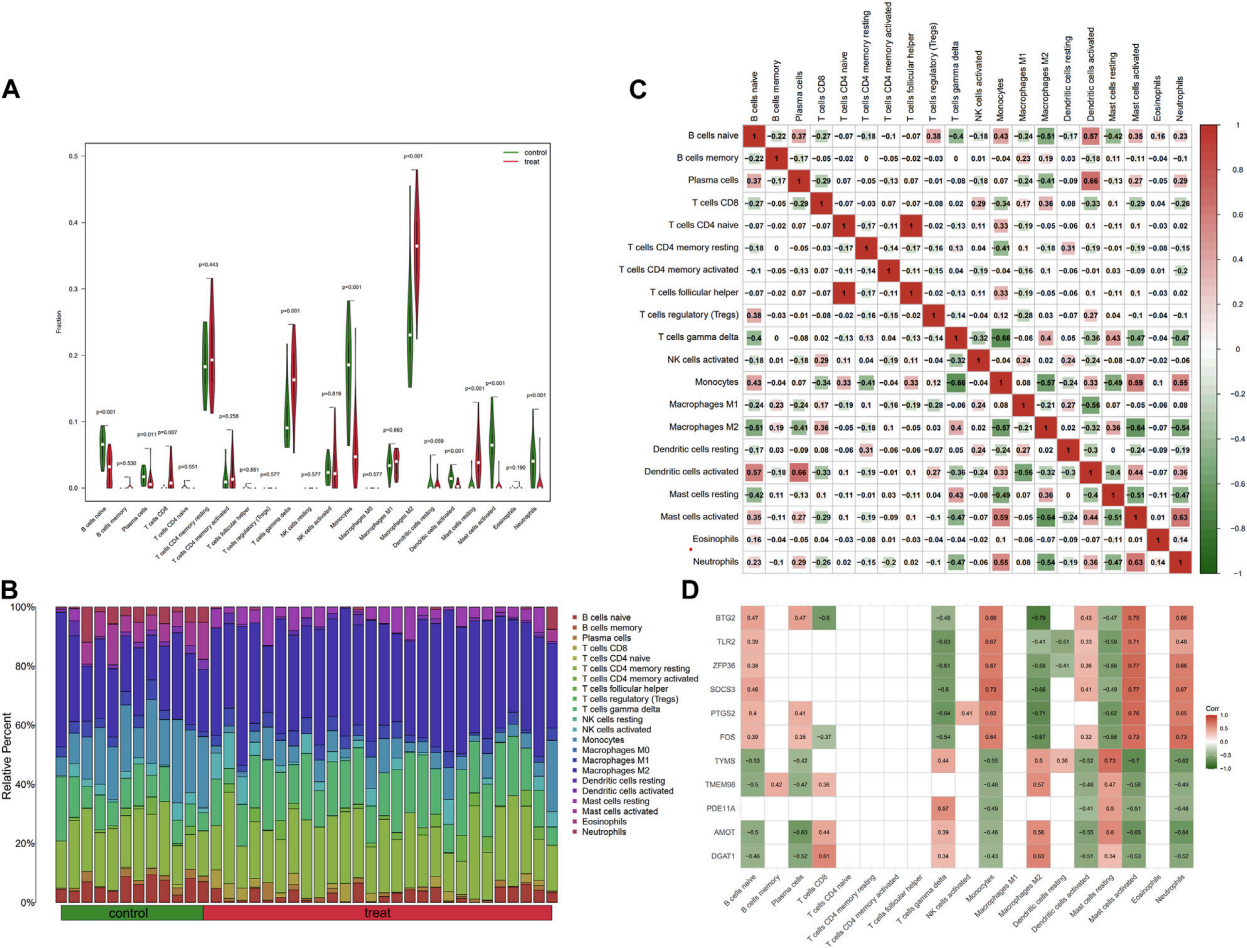
Results of CIBERSORT analysis of GSE89632 (NAFLD-related array). **(A)** Violin plot illustrating immune cell proportions in both groups. The green and fusiform portions on the left denote the HC group, while the red and fusiform portions on the right indicate the NAFLD group. **(B)** Cumulative histogram showing immune cell infiltration. **(C)** Correlation matrix detailing the degree of immune cell infiltration. Red signifies a positive correlation between two immune cells; blue represents a negative correlation. The larger the numerical value, the stronger the correlation—either positive or negative. **(D)** Correlation matrix showing the degree of infiltration of immune cells and hub genes. Red indicates a positive correlation, and blue signifies a negative correlation. NAFLD, nonalcoholic fatty liver disease; HC, healthy control.

Using the CIBERSORT algorithm, we analyzed the DEGs in liver samples between HC and NAFLD patients. Distinct differential expressions of immune fractions in the liver were observed in NAFLD patients (Figures 4A, B). In the context of NAFLD patients, based on the results derived from the two figures above, CD8 T cells, γδ T cells, M2 macrophages, and activated mast cells were increased in the liver. Conversely, naïve B cells, plasma cells, monocytes, activated dendritic cells, activated mast cells, and neutrophils were decreased. Meanwhile, γδ T cells and M2 macrophages were negatively associated with monocytes and active mast cells (Figure 4C). We further analyzed the correlation between validated genes and immune cells. As illustrated in Figure 4D, in liver samples, CD8 T cells, γδ T cells, and M2 macrophages were associated positively with DGAT1, AMOT, PDE11A, TYMS, and TMEM98. Meanwhile, γδ T cells and M2 macrophages were negatively associated with FOS, PTGS2, SOCS3, ZFP36, TLR2, and BTG2.

### 3.6 Molecular docking studies

**FIGURE 5:**
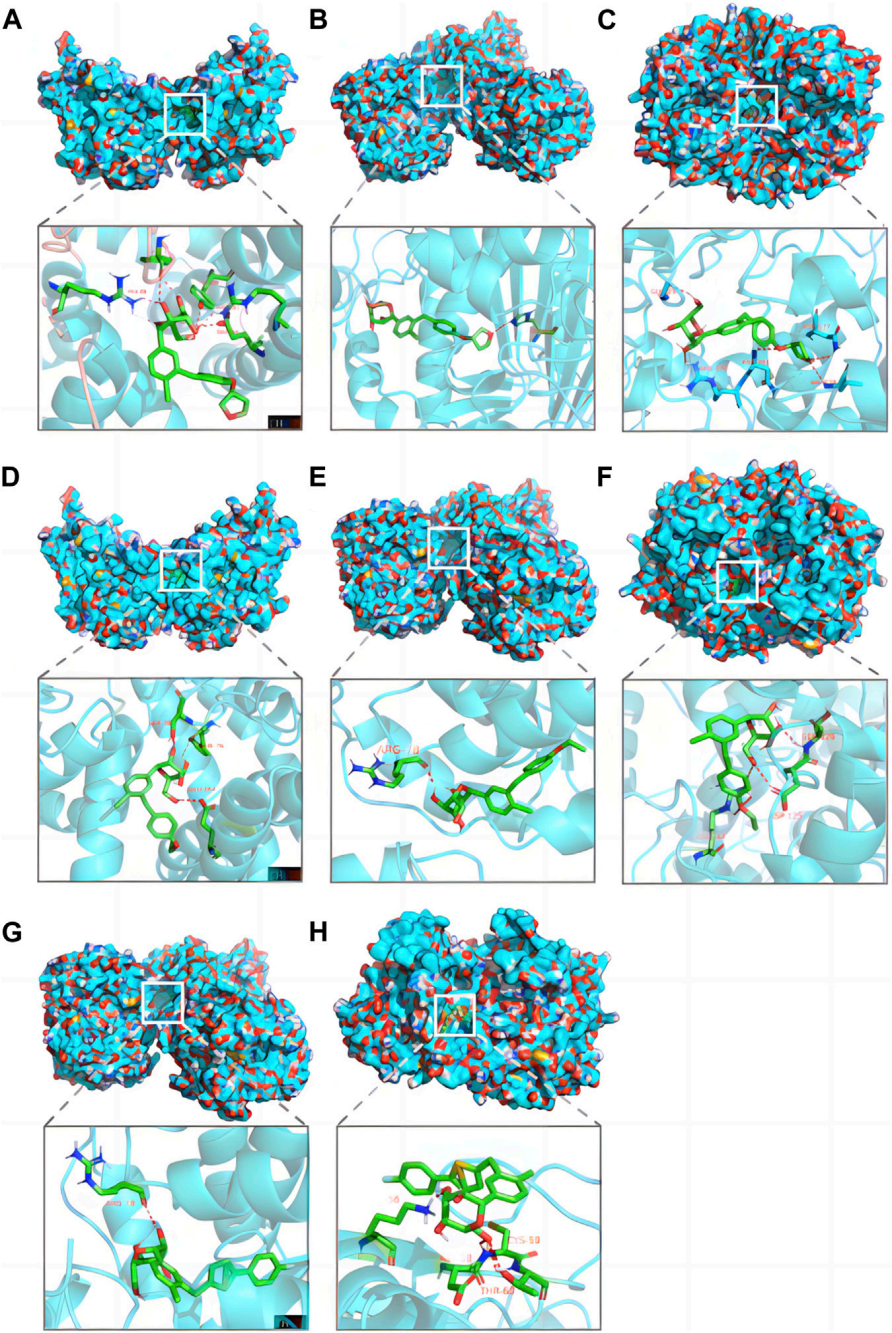
Pharmacological targets of SGLT2 inhibitors in NAFLD-related AF. Molecular docking revealed the binding of empagliflozin to DGAT1 **(A)**, TYMS **(B)** and PTGS2 **(C)**; dapagliflozin to DGAT1 **(D)**, TYMS **(E)** and PTGS2 **(F)**; canagliflozin to TYMS **(G)** and PTGS2 **(H)**. AF, atrial fibrillation; NAFLD, nonalcoholic fatty liver disease.

Screening with TargetNet revealed potential interactions between SGLT2i and the experimentally validated proteins DGAT1, TYMS, and PTGS2, suggesting a connection between NAFLD and AF through inflammatory pathways. As presented in Table 2, the average affinity of empagliflozin, dapagliflozin and canagliflozin was −9.3 kcal/mol, −8.4 kcal/mol, and −5.5 kcal/mol, respectively. Figure 5 illustrates the detailed docking process at each key target site. Notably, the larger the absolute value of the docking affinity, the more stable the binding. Thus, we hypothesize that empagliflozin will have an advantage over dagliflozin and cargliflozin. Consequently, these findings propose empagliflozin as a potential therapeutic approach.

**TABLE 2:** Molecular docking results.

Compound	Target	PDB	Energy (kcal/mol)
empagliflozin	*DGAT1*	6VYI	−9.2
empagliflozin	*TYMS*	6ZXO	−9.4
empagliflozin	*PTGS2*	5F19	−9.4
dapagliflozin	*DGAT1*	6VYI	−8.6
dapagliflozin	*TYMS*	6ZXO	−8.6
dapagliflozin	*PTGS2*	5F19	−8.1
canagliflozin	*DGAT1*	6VYI	0
canagliflozin	*TYMS*	6ZXO	−9.9
canagliflozin	*PTGS2*	5F19	−6.5

PDB, protein data bank.

## 4 Discussion

In this study, we identified eight co-upregulated and 31 co-downregulated DEGs in the two datasets. Through validation of the upregulated genes and hub genes identified by PPI analysis of the co-downregulated DEGs, we found that AMOT, PDE11A, TYMS, TMEM98, and PTGS2 exhibited good diagnostic potential for NAFLD-related AF. CIBERSORT analysis revealed elevated levels of CD8 T cells, γδ T cells, and M2 macrophages in the livers of NAFLD patients, linking systemic inflammation to NAFLD and AF. Additionally, several hub genes like DGAT1, TYMS, and PTGS2 closely related to mitochondrial function, suggesting that mitochondrial disturbances may underpin the systemic inflammation in NAFLD, which possibly exacerbating AF. Last but not least, molecular docking analysis revealed a strong binding affinity between empagliflozin and DGAT, TYMS, and PTGS2, suggesting the potential of empagliflozin as a therapeutic option for NALFD-related AF.

The concurrent rise in the prevalence of NAFLD and AF in recent times has prompted considerable research interest. NAFLD is characterized by an inflamed liver state that can potentially trigger AF (Chen et al., 2021). This highly active hepatic condition induces various pro-inflammatory cytokines, which can infiltrate the heart, leading to immune cell infiltration and inflammation—key elements of the liver-heart axis that could culminate in AF (Peiseler et al., 2022). Upon reviewing the literature, we have collectively validated five co-upregulated DEGs (DGAT1, AMOT, PDE11A, TYMS, and TMEM98) that were elevated in patients with AF, NAFLD, and in those with NAFLD-related AF compared to the HC group. Notably, the latter four genes were particularly elevated in patients with NAFLD and concurrent AF. The DGAT1 gene encodes a protein functioning as a key metabolic enzyme. Extensive research corroborates that DGAT1 exacerbates lipid storage in hepatocytes (de la Rosa Rodriguez et al., 2021), and drugs targeting this protein can reduce liver fibrosis and inflammation in a murine NASH model (Cheng et al., 2022). Additionally, DGAT1 is essential in maintaining mitochondrial autophagy and metabolic integrity, with studies showing that deferiprone-induced iron depletion—prompting PINK1/PARKIN-independent mitochondrial autophagy—also triggers DGAT1-dependent lipid droplet biogenesis, indicating a synergistic interaction between metabolic reprogramming and mitochondrial autophagy following chelation therapy (Glenn et al., 2011; Long and McWilliams, 2023). No studies to date have established a link between DGAT1 and AF. However, most studies have shown that DGAT1 is associated with inflammation. Huang et al. suggest that DGAT1 inhibitors can protect pancreatic β cells from palmitate-induced apoptosis (Huang et al., 2021). While Graham et al. propose that DGAT1 can mediate autoimmune encephalomyelitis by inhibiting the formation of retinol-dependent regulatory T cells (Graham et al., 2019). Some studies also indicate that DGAT1-dependent triglyceride storage in macrophages can protect mice from diet-induced obesity insulin resistance and inflammation (Koliwad et al., 2010). The AMOT gene belongs to the angiomotin family of angiostatin-binding proteins, which may mediate the inhibitory effect of angiostatin on angiogenesis, and no research thus far has associated this gene with AF or NAFLD. AMOT plays a crucial role in regulating Hippo pathway activity, which is implicated in lipid metabolism, inflammation, and fibrogenesis, all processes intrinsic to the pathogenesis of NAFLD (Zhang et al., 2010). The Hippo pathway also relates to cardiac development, repair, and homeostasis, and its dysregulation can lead to myocardial hypertrophy, fibrosis, and dysfunction, known risk factors for AF (Ikeda et al., 2019). PDE11A is an enzyme-degrading cyclic nucleotide (cAMP and cGMP), and no studies have associated this gene with AF or NAFLD to date. However, research indicates that the cAMP/PKA signaling pathway is a potential therapeutic target in metabolic diseases, including obesity, type 2 diabetes, and related complications (London and Stratakis, 2022). TYMS encodes thymidylate synthase, which is crucial for DNA replication and repair. While the relationship between TYMS and cancer has been extensively studied (Zhao and Kaldis, 2023), its role in NAFLD, AF, or inflammation requires further exploration. TYMS, crucial for maintaining deoxythymidine triphosphate (dTTP) levels within mitochondria for accurate DNA replication, may play a role in NAFLD-AF pathogenesis through its impact on cellular and mitochondrial integrity (Anderson et al., 2011). The TMEM98 gene encodes a transmembrane protein. Intriguingly, it can also be secreted via exosomes. Under antigen-non-specific and antigen-specific Th1-skewing conditions, eukaryotic recombinant TMEM98 promotes Th1 cell differentiation and stimulates IFNγ secretion (Fu et al., 2015). Although there is no direct connection with AF or NAFLD yet, transmembrane proteins are often crucial in biological processes and disease states due to their role in cell signaling, transport, and communication, meriting further research.

We also identified 31 co-downregulated genes and selected the six most central genes for validation (FOS, PTGS2, SOCS3, ZFP36, TLR2, and BTG2). Ultimately, only PTGS2 demonstrated substantial diagnostic value for patients with NAFLD in conjunction with AF. Enrichment analysis of the 31 downregulated genes reveals a downregulation of the NF-κB and TNF pathways, which seems contrary to our understanding of NAFLD and AF’s association with chronic systemic inflammation. However, enriched within these two pathways are genes such as PTGS2, a cyclooxygenase that serves as a key enzyme in prostaglandin biosynthesis. Although COX2 (PTGS2) is usually considered to induce inflammation, previous studies have found that COX-2 deficient mice, or those treated with COX inhibitors, display exaggerated inflammatory responses in the lungs, leading to the development of asthma (Peebles et al., 2002). PTGS2, also known to modulate mitochondrial function and lipid metabolism pathways, plays a crucial role in ferroptosis during cardiac and pulmonary disease processes, challenging the traditional view of its sole pro-inflammatory role (Chen et al., 2022; Lv et al., 2023). These pathways are also enriched with SOCS3 (suppressor of cytokine signaling 3), an essential molecule for negatively regulating cellular signaling pathways, by inhibiting inflammatory cytokines such as interleukin-6 (IL-6) and interferon (IFN) (Zhang et al., 2020). Thus, SOCS3 downregulation could exacerbate inflammation. Additionally, SOCS3 can affect the differentiation of immune cells, particularly regulating the Th1/Th2 balance of T cells and the polarization of macrophages towards the M2 phenotype, thereby influencing the course of immune responses (Huang W. et al., 2022). Recent studies have found an increase in SOCS3 expression in the hepatocytes of patients with NAFLD, which may be related to inflammation and insulin resistance in these conditions (Zhang et al., 2021). Conversely, miR-455-5p mimics can accelerate the progression of AF by activating the STAT3 signaling pathway through direct binding to SOCS3 (Li et al., 2021). SOCS3 not only modulates cellular signaling by inhibiting inflammatory cytokines but also interacts with proteins like KEAP1 to directly regulate mitochondrial-induced apoptosis, and may indirectly affect mitochondrial function by influencing the stability and activity of NRF2, underscoring its significance as a potential target for research and therapeutic intervention (Li et al., 2022; Meng et al., 2023). Herefore, even if these two genes are enriched in downregulated NF-κB and TNF pathways, this does not necessarily signify a downregulation of inflammation. Given these insights, further study into these genes and pathways is certainly warranted.

**FIGURE 6:**
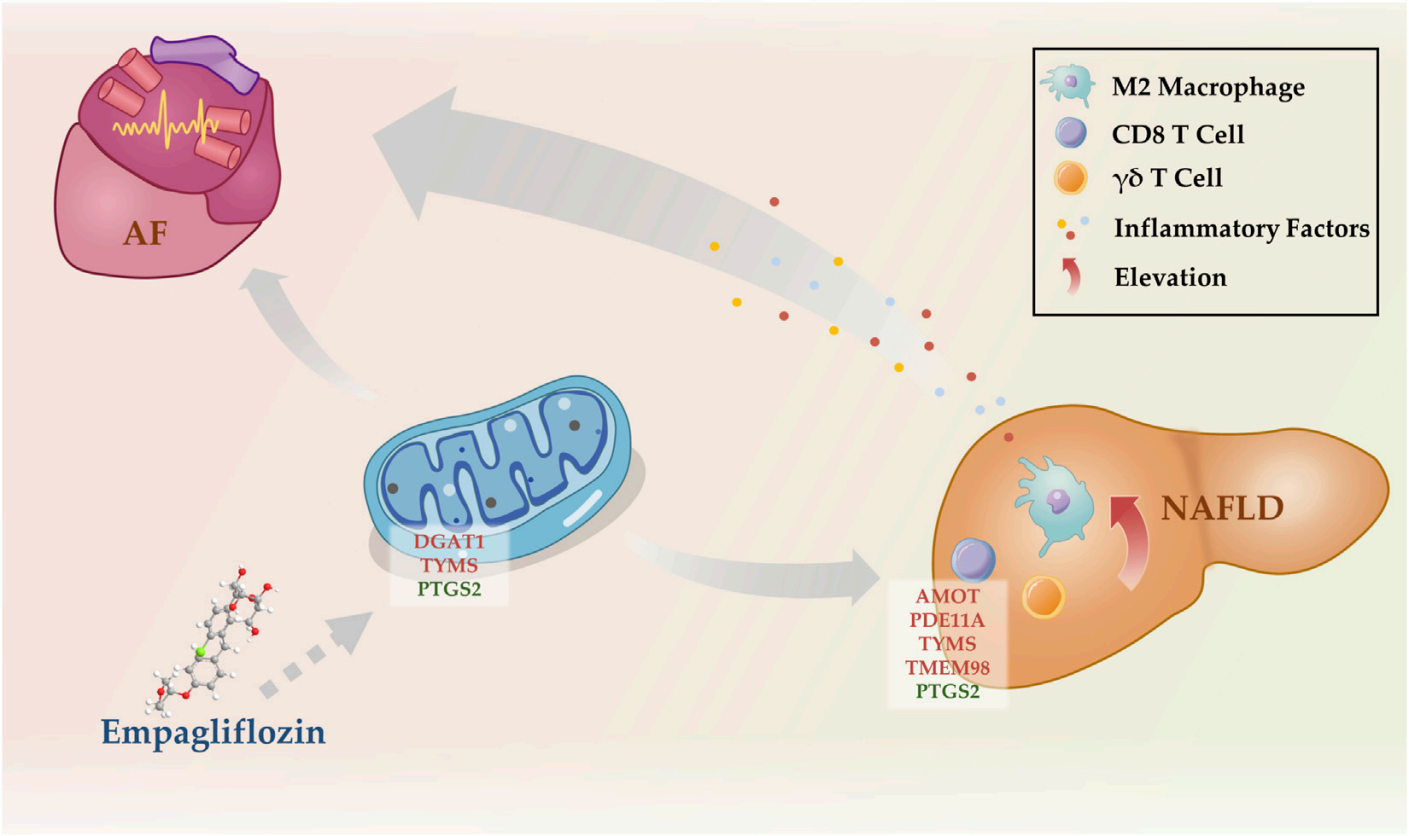
Potential mechanisms by which NAFLD promotes the development of AF. NAFLD, nonalcoholic fatty liver disease; AF, atrial fibrillation.

Mitochondrial dysfunction and systemic inflammation may bridge the gap between NAFLD and AF. Literature and our previous study found that mitochondrial dysfunction is involved in the pathogenesis of NAFLD and AF (Chen et al., 2021; Liu et al., 2021). Under metabolic stress conditions, ROS production increases in mitochondria, and once ROS levels exceed the neutralization capacity of its own antioxidant system, it damages the mitochondrial membrane and mitochondrial DNA, causing mitochondrial dysfunction and inducing hepatic inflammation via the nuclear factor-κB (NF-κB) and nucleotide-binding oligomerization domain-like receptor family pyrin domain-containing 3 (NLRP 3) pathways (Chen et al., 2020). When cardiomyocyte mitochondrial dysfunction occurs, ΔΨm decreases, ATP production decreases, leading to the opening of membrane ATP-sensitive potassium channels (sarcKATP), slowing of myocardial localized electrical activity conduction, increasing inhomogeneity, and ease of formation of refractoriness, which induces AF (Lkhagva et al., 2021). In addition, myocardial mitochondria store a large amount of Ca2+, which is a reservoir of intracellular Ca2+, and when mitochondrial dysfunction occurs, the intracellular calcium balance is disturbed, which promotes the occurrence of AF (Liu et al., 2021). It is believed that NAFLD potentially contribute to structural, functional, electrical, and autonomic remodeling of the heart by induce metabolic disorders, inflammation, oxidative stress and promote epicardial adipose tissue (EAT) expansion, which ultimately leading to increased susceptibility to AF (Chen et al., 2021). Inflammation and immune activation are important bases for the development of AF (Coppini et al., 2019). In our study, we revealed an elevation in the populations of CD8 T cells, γδ T cells, and M2 macrophages in the liver of NAFLD patients through CIBERSORT analysis. This information sheds light on the immunological landscape of NAFLD, potentially linking liver inflammation with the systemic inflammatory state observed in these patients. CD8 T cells, also known as cytotoxic T cells, are typically associated with the direct killing of infected or transformed cells. However, in the context of NAFLD, their elevation may contribute to the hepatic inflammatory environment that characterizes this disease, possibly through the secretion of pro-inflammatory cytokines such as TNF-alpha and interferon-gamma (Sutti and Albano, 2020). Niema and others, through fluorescence-activated cell sorting, demonstrated that CD8 + CD28null T lymphocytes are a potent and independent predictive factor for the occurrence of postoperative atrial fibrillation (POAF) after elective cardiac surgery (Kazem et al., 2020). As an unconventional subset of T cells, γδ T cells possess innate-like characteristics and can rapidly produce a variety of cytokines in response to pathogenic or inflammatory stimuli (Lee et al., 2014). Further, certain studies analyzing atrial samples from patients with permanent AF using CIBERSORT have revealed a significant increase in the proportion of γδ T cells (Stone et al., 2021). M2 macrophages, known for their roles in wound healing and tissue repair, have been implicated in the progression of NAFLD, particularly in the development of fibrosis. Their increased numbers may reflect an attempt to control liver inflammation and repair tissue damage, but could also contribute to fibrosis via the production of pro-fibrotic factors (Wei et al., 2022). Research by Chris and his team found an association between the increase in pro-fibrotic M2 macrophages in human atrial tissue and an elevated expression of pre-collagen and anti-fibrotic BNP genes (Watson et al., 2020). Building upon these findings, we propose that the immune cells in NAFLD could release a plethora of inflammatory mediators into the circulation, fostering a state of systemic chronic inflammation. This inflammatory milieu could potentially create a pro-arrhythmic environment, predisposing individuals with NAFLD to the development of AF (Figure 6). In a word, NAFLD promotes AF development as a result of multiple mechanisms, in which mitochondrial dysfunction and systemic inflammation play an important role, but the specific mechanisms need to be explored in more subsequent studies.

Both NAFLD and AF are metabolic-related disorders, underscoring the possibility of intervention through metabolic modulation. Recent studies have revealed that the beneficial effects of SGLT2i on NAFLD seem to be achieved by directly modulating multiple processes, including endoplasmic reticulum stress, oxidative stress, low-grade inflammation, autophagy, and cell apoptosis (Nasiri-Ansari et al., 2021; Feijóo-Bandín et al., 2022; Radlinger et al., 2023). These effects have been confirmed *in vitro* studies, animal models, and clinical trials (Androutsakos et al., 2022; Feijóo-Bandín et al., 2022). Moreover, real-world studies have found that SGLT2i can reduce the risk of new-onset AF in patients with T2DM and improve clinical benefits in patients with T2DM and AF (Zinman et al., 2015; Zelniker et al., 2020; Hsiao et al., 2022). Randomized controlled trial findings also suggest that SGLT2i can decrease the recurrence rate after AF ablation (Kishima et al., 2022; Zhao et al., 2023). In this study, empagliflozin has been shown to bind well with DGAT1, TYMS, and PTGS2. This suggests a potential avenue for therapeutic interventions targeted at these molecular interactions in the context of NAFLD and AF. Prior studies have indicated that SGLT2i could reduce renal lipid deposition through the inhibition of DGAT1 (Wang et al., 2017). This mechanism may also provide a promising therapeutic approach for NAFLD, given the central role of lipid accumulation in the pathogenesis of this disease. Concurrently, an effective reduction in lipid accumulation could indirectly attenuate the onset or progression of AF, considering the shared metabolic underpinnings of these conditions (Lavie et al., 2017). While there is currently no research specifically examining the interaction of empagliflozin with TYMS and PTGS2, these molecules represent potential therapeutic targets for managing NAFLD and NAFLD-related AF. Given that DGAT1, TYMS, and PTGS2 all play roles in mitochondrial function, the ability of SGLT2 inhibitors like empagliflozin to interact with these molecules suggests they might also exert beneficial effects on NAFLD and AF by modulating mitochondrial dysfunction. While current literature on the direct impact of SGLT2i on mitochondrial function is limited, the implication of these interactions indicates a potential pathway where SGLT2i could improve AF outcomes by addressing mitochondrial dysfunction. Further investigation is required to elucidate this mechanism and its clinical relevance.

This study possesses certain limitations. Firstly, the sample size used for the AF bioinformatic analysis was small. Secondly, the samples we used for analysis and validation were all peripheral blood from patients with AF, not atrial tissue as it's hard to get. Thirdly, the software tools we used for molecular docking studies used simplified models that do not explicitly account for water molecules, ion concentrations, or pH values in the docking process, and can not get the information about their interaction sites, and more advanced simulations that can take more factors into account are needed in the future. Lastly, the absence of animal experiments in our study design presents another limitation. Thus, the role of hub genes and mechanisms needs to be further identified through *in vitro* or *vivo* study.

## 5 Conclusion

In summary, we firstly discovered that AMOT, PDE11A, TYMS, TMEM98, and PTGS2 are associated with NAFLD-related AF and hold strong diagnostic values. Our study also indicates that mitochondrial dysfunction and inflammation may be potential mechanisms bridging NAFLD and AF. Additionally, we identified empagliflozin as a potentially effective therapeutic agent for NAFLD-related AF at the molecular structure level. These novel insights contribute to the further understanding, diagnosis, and intervention of NAFLD-related AF.

## Data Availability

The datasets presented in this study can be found in online repositories. The names of the repository/repositories and accession number(s) can be found in the article/Supplementary Material.
