# Supplementary material for: Identification of key genes increasing susceptibility to atrial fibrillation in nonalcoholic fatty liver disease and the potential mechanisms: mitochondrial dysfunction and systemic inflammation
Source: Front Pharmacol. 2024 Mar 14;15:1360974. doi: 10.3389/fphar.2024.1360974 (PMC10972919; doi:10.3389/fphar.2024.1360974)
Supplement: Supplementary file 3 [file Table3.DOCX]

**Supplementary Table 1.** Primers used in this study for RT-qPCR experiments

| **Gene** |  | **Forward (5' to 3')）** | **Size（bp）** |
| --- | --- | --- | --- |
| Homo *DGAT1* | F | GCGGTGGGTGCCCTGACG | 159 |
|  | R | CTTGAGGAAGAGGATGGTGTGCG |  |
| Homo *RHOBTB1* | F | TCTTGGAGCGTTCTCGGGATG | 199 |
|  | R | GGGCAAAAGTGCTTGATTTCTGG |  |
| Homo *AMOT* | F | TATTTGGAGGAGAATGTGATGAGAC | 93 |
|  | R | AGGAGAGTGACTGATGACTGTTGTG |  |
| Homo *PDE11A* | F | TTTGACAGTGAGGAAAAACTATCGG | 115 |
|  | R | CAGAATGTCTTGAAACCCAGCAG |  |
| Homo *FOS* | F | GATGTTCTCGGGCTTCAACGCAG | 200 |
|  | R | AGATGGCAGTGACCGTGGGAATG |  |
| Homo *PTGS2* | F | AAACCGAGGTGTATGTATGAGTGTG | 170 |
|  | R | ATCCCTTGAAGTGGGTAAGTATGTAG |  |
| Homo *SOCS3* | F | GTCCCCCCAGAAGAGCCTATTAC | 109 |
|  | R | TTCCGACAGAGATGCTGAAGAGT |  |
| Homo *MYC* | F | TCCCCTACCCTCTCAACGACAG | 204 |
|  | R | CGATTTCTTCCTCATCTTCTTGTTCC |  |
| Homo *TYMS* | F | AGGCATTTTGGGGCAGAATACAG | 101 |
|  | R | TCAGGGTTGGTTTTGATGGTGTC |  |
| Homo *TMEM98* | F | CCAGGGTGGATGATGTTGTGAAG | 128 |
|  | R | CAGATGGCAGGCATTCCTTGTC |  |
| Homo *ZFP36* | F | CTTCCACTGTCACCCTCTGCCTTC | 118 |
|  | R | GCCCCCAGACGCTGATAGGAG |  |
| Homo *TLR2* | F | CTTCTCCCATTTCCGTCTTTTTG | 125 |
|  | R | TCTTGGTGTTCATTATCTTCCGC |  |
| Homo *BTG2* | F | CTCACAGAGCACTACAAACACCACT | 135 |
|  | R | GGGCTGGCTGAGTCCGAT |  |
| Homo *GAPDH* | F | GAAGGTGAAGGTCGGAGTC | 227 |
|  | R | GAAGATGGTGATGGGATTTC |  |


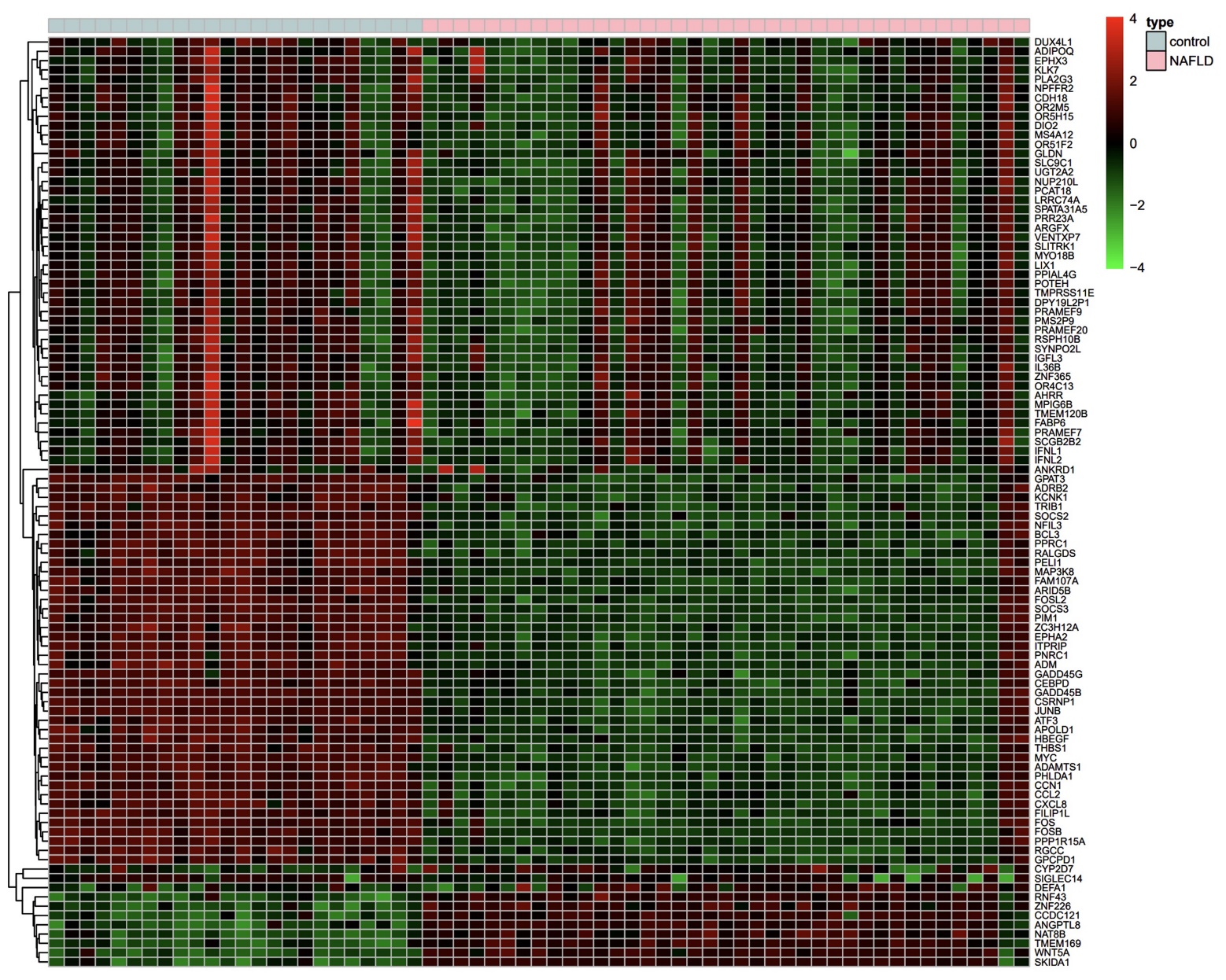


**Supplementary Figure 1.** Hierarchical clustering analysis of NAFLD-related top 50 up-regulated and top 50 down-regulated expressed genes. NAFLD: non-alcoholic fatty liver disease.


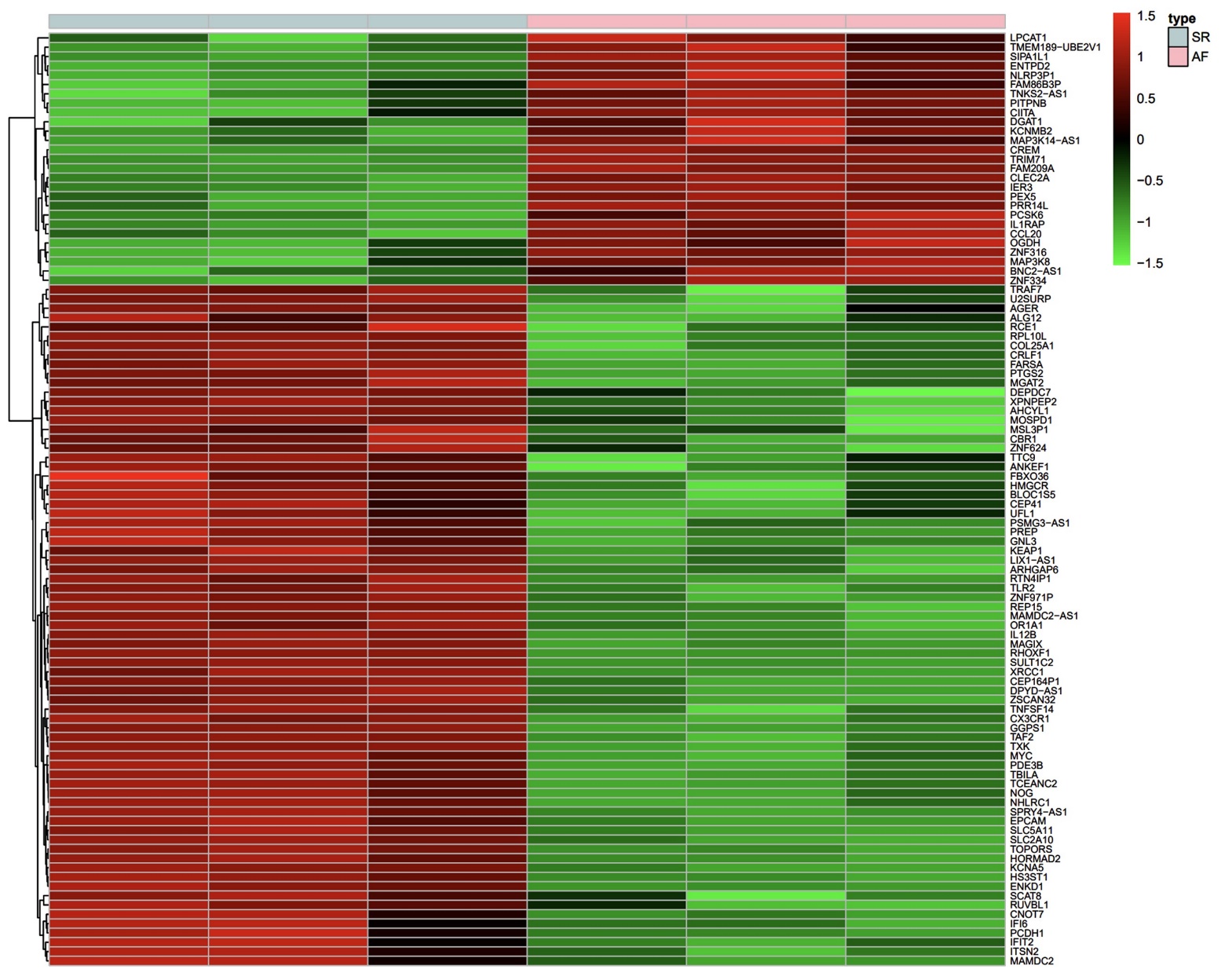


**Supplementary Figure 2.** Hierarchical clustering analysis of AF-related top 50 up-regulated and top 50 down-regulated expressed genes. AF: atrial fibrillation.


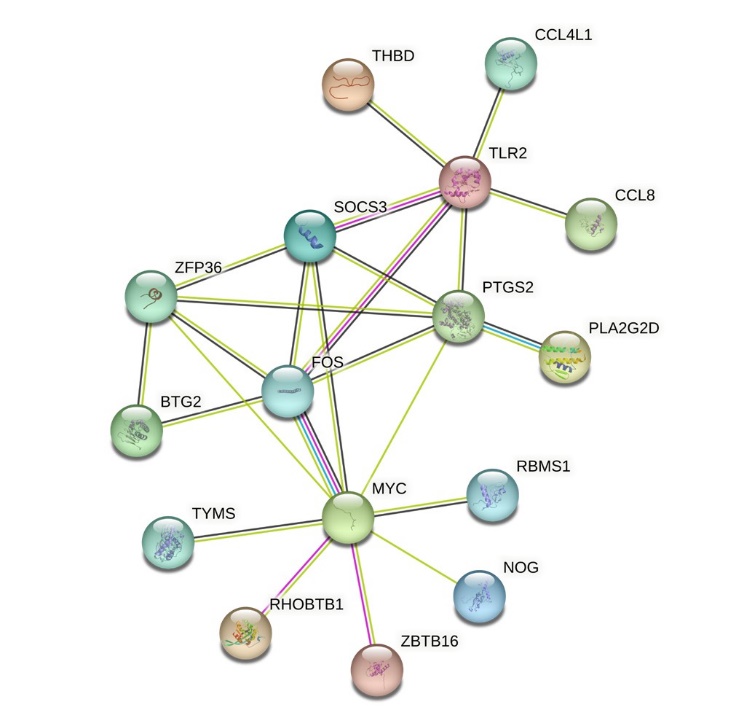


**Supplementary Figure 3.** PPI networks constructed by STRING database for co-DEGs (threshold > 0.4). PPI:Protein–protein interaction; DEG: differentially expressed genes.
